# Prostate Cancer: Epigenetic Alterations, Risk Factors, and Therapy

**DOI:** 10.1155/2016/5653862

**Published:** 2016-11-06

**Authors:** Mankgopo M. Kgatle, Asgar A. Kalla, Muhammed M. Islam, Mike Sathekge, Razia Moorad

**Affiliations:** ^1^Division of Hepatology and Liver Research, Department of Medicine, Faculty of Health Sciences, University of Cape Town and Groote Schuur Hospital, Observatory, Western Cape 7925, South Africa; ^2^Division of Rheumatology, Department of Medicine, Faculty of Health Sciences, University of Cape Town and Groote Schuur Hospital, Observatory, Western Cape 7925, South Africa; ^3^Department of Integrative Biomedical Sciences, Institute of Infectious Disease and Molecular Medicine, Faculty of Health Sciences, University of Cape Town, Observatory, Western Cape 7925, South Africa; ^4^Department of Nuclear Medicine, University of Pretoria and Steve Biko Academic Hospital, Private Bag X169, Pretoria, Gauteng 0001, South Africa; ^5^Department of Surgery, Faculty of Health Science, University of Cape Town and Groote Schuur Hospital, Observatory, Western Cape 7925, South Africa

## Abstract

Prostate cancer (PCa) is the most prevalent urological cancer that affects aging men in South Africa, and mechanisms underlying prostate tumorigenesis remain elusive. Research advancements in the field of PCa and epigenetics have allowed for the identification of specific alterations that occur beyond genetics but are still critically important in the pathogenesis of tumorigenesis. Anomalous epigenetic changes associated with PCa include histone modifications, DNA methylation, and noncoding miRNA. These mechanisms regulate and silence hundreds of target genes including some which are key components of cellular signalling pathways that, when perturbed, promote tumorigenesis. Elucidation of mechanisms underlying epigenetic alterations and the manner in which these mechanisms interact in regulating gene transcription in PCa are an unmet necessity that may lead to novel chemotherapeutic approaches. This will, therefore, aid in developing combination therapies that will target multiple epigenetic pathways, which can be used in conjunction with the current conventional PCa treatment.

## 1. Introduction

According to the World Cancer Research Fund's list, South Africa is ranked 50th among countries with the highest cancer prevalence [1]. Recent article published in Lancet projected cancer to become a leading cause of deaths in South Africa, with an increase from 77 400 cases in 2008 to 112 921 new cases in 2030 [2]. Prostate cancer (PCa) is the most prevalent urological cancer and one of the four top cancers including Kaposi sarcoma and colorectal and lung cancers, which affect aging men in South Africa [2]. It is estimated that 1 in 8 South African aging men will develop PCa at some point in their lifetime. The most common type of PCa is acinar adenocarcinoma, representing 90% of the PCa cases [3].

Prostate is a walnut sized organ located just between the bladder and penis, and it slowly grows larger to an average weight of 40 grams in aging men. A prostate gland surrounds the urethra that empties urine from the bladder and also secretes prostate fluid that protects sperm. These physiological functions may be compromised during various prostate diseases including prostatitis, benign prostatic hyperplasia or hypertrophy, and cancer. PCa begins when aberrant semen-secreting prostate gland cells develop and proliferate uncontrollably. If left untreated, PCa may metastasise to other parts of the body, particularly to the lymph nodes and bones. Although most patients may remain asymptomatic in the early stages, advanced PCa may be accompanied by a variety of urinary symptoms including troublesome nocturia, dysuria, hematuria, hematospermia, pain and swelling in the legs and pelvic area, loss of bladder, and erection control. On the other hand, benign prostatic hyperplasia or hypertrophy may show similar symptoms but is rarely life-threatening. In this case, the use of total plasma prostate specific antigen (tPSA) level becomes important. According to ASC, a tPSA level of more than the cut-off value of 4 ng/mL may indicate the occurrence of PCa [4]. To overrule the possibility of an elevated tPSA level in a subset in nonmalignant conditions, transrectal biopsy guided by ultrasound can be used to validate the diagnosis [4, 5].

Owing to its slow growth, PCa may take up to 10 years to progress from precursor prostatic intraepithelial neoplasia (PIN) to an invasive carcinoma. Histologically, PIN can be classified into low and high grade, which are generally characterised by various molecular or cellular architecture [6]. The Gleason grading system developed by Dr. Donald F. Gleason between 1966 and 1974 and recently reviewed and improved by the 2014 International Society of Urological Pathology remains one of the most powerful predictors for the prognostic outcome of PCa. An alternative to the current Gleason score was recently proposed with the potential to lessen the overtreatment of low-grade PCa detected by PSA screening. The new simplified PCa grading incorporates five grades, and these include grade group 1 (Gleason score ≤ 6), grade group 2 (Gleason score 3 + 4 = 7), grade group 3 (Gleason score 4 + 3 = 7), grade group 4 (Gleason score 8), and finally grade group 5 (Gleason score 9-10) [7].

PCa is a heterogeneous disease, and its occurrence differs extremely from patient to patient even within the same tumour. The great disparity in the PCa architecture and incidence rates may be attributable to genomic instabilities and alterations associated with various PCa risk factors. Therefore, this review aims to provide a comprehensive understanding of the prostate carcinogenesis-related epigenetic signatures and their mediators. In addition, the associated PCa risk factors and recent treatment options are discussed.

## 2. Current Prostate Cancer Treatment

Depending on the severity of the disease, current therapies for PCa may include watchful waiting, hormone therapy, therapeutic vaccines, bone-directed treatment, cryotherapy, radiation therapy, and surgery. Although these treatment options may improve the quality of life of patients by significantly delaying or inhibiting the progression of the disease, chemotherapeutic resistance commonly develops often resulting in death [8, 9]. In addition, an estimated 30% of patients relapse following the initial treatment. The 5-year survival rate for the localised and regional PCa is nearly 100%; however, this percentage drops to 28% in cases where cancer has metastasised to distant organs [9, 10].

**Figure 1:**
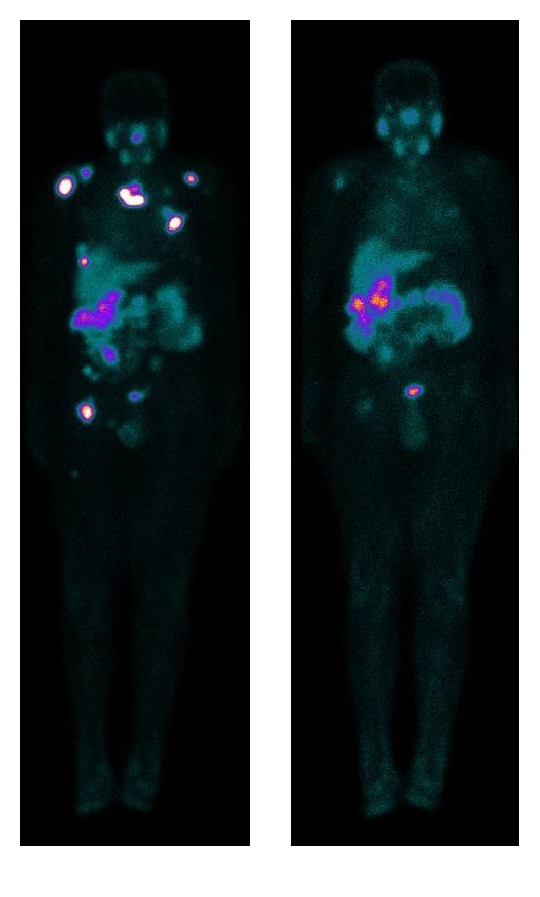
Anterior whole body ^177^Lu-PSMA scan of a 72-year-old patient with metastasised castration-resistant prostate cancer. This demonstrates normal biodistribution in the nasal region, salivary glands, liver, and spleen gastrointestinal and urinary system, with multiple PSMA-avid metastases in the prostate bed, clavicle, sternum, clavicle, vertebrae, iliac bone, and femora. An excellent response to therapy was observed after 3 cycles of ^177^Lu-PSMA-RLT with decrease in serum PSA level (from 63 to 4.25 ng/mL).

Two recently approved hormone therapy agents, the AR antagonist enzalutamide and the CYP17A1-inhibitor abiraterone, have proven to be well tolerated and effective in many metastasised castration-resistant PCa patients [11]. Nonetheless, these therapy agents remain noncurative, suggesting an urgent need of novel systemic treatment that can improve the overall survival of PCa patients. This encouraged the development of novel therapeutic approaches such as the prostate specific membrane antigen-targeted radioligand therapy (PSMA-RLT) [12]. PSMA-RLT allows the delivery of high dose of therapeutic radiation to cancer cells, while lessening the exposure of normal cells. PSMA, also known as folate hydrolase I or glutamate carboxypeptidase II, is a cell surface protein that is significantly overexpressed in refractory or metastasised castration-resistant PCa cells. The PSMA-RLT involves binding of a ligand PSMA to a radioactive isotope Lu-177, and this facilitates the detection of metastatic sites for treatment as illustrated in Figure 1. The widely used radiopharmaceutical is ^177^Lu-PSMA. The radionuclide is transported directly to the tumour cells and radioactive decay of ^177^Lu emits beta particles, which are absorbed by the disease targeted sites thereby damaging the surrounding PCa cells [13, 14]. PSMA-RLT with ^177^Lu-PSMA holds a great promise to be a magic bullet for patients with metastasised castration-resistant PCa with appropriate selection and follow-up by ^68^Ga-PSMA PET/CT as it follows a theranostic approach, which is the basis for successful radionuclide therapy [14–16].

Radiomics is a high throughput approach used to extract a large number of qualitative and quantitative features from medical images, which could improve tumour phenotype characterisation and treatment outcome prediction [17]. Positive ^68^Ga-prostate-specific membrane antigen for diagnostics with positron emission tomography/computed tomography (PET/CT) has the ability to capture intratumoural heterogeneity in a noninvasive way as well as to assess the response to PSMA-RLT. This imaging has brought radiomics closer to reality when it significantly reduced false-negative biopsies by guiding the needle to the highest Gleason score lesion in a comparative trial of biopsied PCa patients [18]. ^68^Ga-PSMA PET/CT imaging and the use of PSMA-RLT with ^177^Lu-PSMA may provide better risk stratification of PCa patients, if the findings are complemented with the knowledge of somatic mutations and epimutations. Recent data on a mouse model have shown that combining epigenetic drugs with immunomodulatory antibodies that target cytotoxic T-lymphocyte antigen 4 (CTLA-4) and programmed cell death 1 (PD-1) improves antitumor activity compared to the case when they were used individually [19]. For instance, syngeneic mammary and colorectal carcinoma models demonstrated that combination of 5-azacytidine (DNA demethylating agent) plus entinostat (histone deacetylase inhibitor) and CTL-4 plus CD-1 significantly improved therapeutic outcomes. The same study have also shown that anti-CD40 plus anti-CD137 immunotherapy combined with panobinostat (another histone deacetylase inhibitor) delayed tumour growth in syngeneic mammary (i.e., 4T1.2), colon (i.e., CT26), and prostate carcinoma mouse models [20].

## 3. Epigenetic Signatures and Mediators in Prostate Cancer

The epigenetic regulation of gene expression has been substantially studied for over 19 years now and is embraced as the well-known hallmark of tumorigenesis. Perturbed key multiple and important cellular processes that are normally suppressed or activated to inhibit malignant transformation is the common phenotype of aberrant gene expression. Cancer-related epigenetics, also known as epimutations, involves anomalous epigenetic alterations of gene transcription in cancer cells that are not attributable to any change in the nucleotide sequence [21, 22]. Advanced research in this rapidly growing and evolving landscape of cancer epigenetics has revealed a large amount of data showing global and specific epigenetic alterations, which are characterised by suppressed cell apoptosis, sustained cell proliferation, and invasion. These cellular manifestations are associated with hijacked or disrupted physiological pathways mediated by inactivating mutations and silencing of the transcriptional activities of cell-cycle activators [23, 24]. Several lines of evidence show that histone modifications, DNA methylation, and noncoding microRNA (miRNA/miR) are the most common epigenetic signatures responsible for the aberrant suppression of gene transcription observed in many malignancies. PCa is also influenced by a combination of genetic and/or epigenetic alterations that are strongly linked to perturbed cellular processes and tumour initiation. However, how these aberrant epigenetic signatures are established still remains poorly understood.

## 4. DNA Methylation 

DNA methylation can be categorised as hypermethylation (increased methylation) and hypomethylation (decreased methylation). It involves chemical attachment of a methyl group to the carbon 5 on the genome, thereby leading to an alteration in gene transcription and normal function [21]. This phenomenon is established and maintained by various active DNA methyltransferase (DNMTs) enzymes including DNMT1, DNMT3A, and DNMT3B [21, 22, 25]. Hypermethylation is one of the best dissected epigenetic alterations in PCa and involves well-known genes that are important in DNA damage repair (*GSTP1* and* MGMT*), apoptosis (*DAPK1*;* RASSF1*), hormonal response (*androgen receptor-AR*,* estrogen receptor-ER, *and* RARβ*), inflammatory responses (*prostaglandin endoperoxide synthase 2*-*PTGS2*), cell-cycle control (*CDKN2A*), and metastases (*Cadherins*,* CD44*, and* tissue inhibitor metallopeptidase* (*TIMP)*) [26]. Like in other malignancies, silence in gene expression is the most common trait associated with promoter hypermethylation in PCa. This is often highly detected in advanced pathologic grades or clinical stages and usually accompanied by cancer development, invasion, and metastasis. More recently, hypermethylation of genes such as* Ephrin-A5 *(*Eph*-*A5*) and* transmembrane protease serine 2* (*TMPRSS2*) has been revealed in PCa. Aberrant hypermethylation of the* Eph5A* CpG promoter regions was observed in advanced PCa patients with a Gleason score of 8.* Eph5A* is one of the Eph family of receptors of tyrosine kinases that are implicated in several human malignancies including PCa and hepatocellular carcinoma [27, 28]. TMPRSS2, an AR signalling downstream gene, is a type II transmembrane protease abundantly expressed in the prostate gland that has been shown to induce tumorigenesis when altered. DNMT1-induced hypermethylation at the promoter region of* TMPRSS2 *was observed in PCa cells. This was accompanied by a suppressed expression of* AR* and* TMPRSS2*, which was restored through treatment with demethylating agent 5-Aza-2′-deoxycytidine [28]. Overexpression of* ERα* is directly proportional to PCa disease progression and promotes oncogenic events such as fusion of* TMPRSS2* and* estrogen-regulated gene *(*ERG*) located on chromosome 21. It was discovered that* TMPRSS2* is fused to either* ERG* or* ETS variant 1 *(*ETV1*) and this frequent event contributes to PCa tumour progression [29–31]. Global DNA hypomethylation was hypothesised to occur later in PCa than CpG island hypermethylation and therefore is more likely to contribute to PCa metastasis than initiation and progression. Genes that are hypomethylated in PCa cells are limited, and these include LINE1, X-inactive specific transcript, plasminogen activator urokinase, and heparanase [32].

Androgen receptors (ARs) play a fundamental role by interacting with testosterone or dihydrotestosterone, which are crucial for the development of the male reproductive system during embryogenesis and sexual development at puberty. In PCa, aberrant hypermethylation of the AR correlates with suppressed gene transcription and increased PCa cell growth and proliferation [33]. A significant difference in methylation profiles that affect genes including caspase 8, CD14, multiple drug resistant 1, and glypican 3 was observed between the AR-resistant and AR-negative cancer cells [34]. Matthew Freedman and his colleagues from Dana-Farber Cancer Institute and Harvard Medical School have recently shown in the human PCa tissues that the AR cistrome undergoes extensive reprogramming, which is an erasure and remodelling of DNA methylation [35]. This phenomenon was accompanied by localisation of forkhead Box A1 (FOXA1) and homeobox B13 (HOXB13) at the programmed AR binding sites, suggesting the implication of transcription factors in establishing aberrant epigenetic reprogramming [35].

The transcription factor E twenty-six-related gene (ERG) mediates epigenetic alterations in the AR cistrome and prime prostate tumorigenesis in a mouse model [36]. Ten-eleven translocation methylcytosine dioxygenase (Tet) proteins 1, 2, and 3 were demonstrated to preserve the unmethylated CpG islands by converting 5-methylcytosine to hydroxymethylcytosine via oxidation induced through interaction and catalytic activities of iron and alpha-ketoglutarate. In PCa, androgen hormone induced global hydroxymethylation silences Tet 2 expression, leading to poor prognosis. This was detected largely among global* FOXA1-*binding sites and inhibited* FOXA1* binding at specific loci, suggesting that alterations in Tet 2-mediated pathway may have a significant implication for treating advanced PCa [37].

## 5. Interaction among Epigenetic Mechanisms in Prostate Cancer

Histones commonly undergo extensive covalent modifications that alter chromatin structure or function, and these include methylation, acetylation, ubiquitylation, and sumoylation. Histone methylation/demethylation and acetylation/deacetylation are extensively studied [38, 39]. Histone acetylation (or deacetylation) involves the addition (or removal) of an acetyl group on lysine (K) residues within the N-terminal tail protruding from the histone core of the nucleosome and is generally linked to gene activation. When methyl groups are added to (or removed from) histone protein amino acids and result in the transcriptional activation or silence, the process is known as histone methylation (or demethylation) [22]. Lysines can be mono-, di-, or trimethylated, and this can be associated with either silence or active in-gene transcription. For instance, methylation on H3K4 and H3K36 is associated with active gene transcription, whereas mono-, di-, and trimethylation on H3K9, H3K27, and H4K20 mark transcriptionally silent chromatin. Histone acetylation/deacetylation and methylation/demethylation are driven by histone acetyltransferases (HATs)/deacetylases (HDACs) and methyltransferases (HMTs)/demethylases [22, 25].

The polycomb group of proteins including polycomb repressive complex 1 (PRC1) and PRC2 are key transcriptional repressor complexes that induce histone methylation to repress* Hox* genes involved in early development and stem cell differentiation. The main complex, PRC2, is composed of four core subunits: embryonic ectoderm development, suppressor of zeste 12, retinoblastoma binding protein 4, and enhancer of zeste 1 (EZH1) or EZH2 [40, 41]. PRC2 silences gene expression through trimethylation of H3K27, which is catalysed by its enzymatic units EZH1 and EZH2 [42, 43]. Disrupted activity of EZH2 through aberrant histone methylation promotes transcriptional instability that favours tumorigenesis and drug resistance. In castration-resistant PCa, overexpression of EZH2 via H3K27me3 is associated with poor clinical outcome, prognosis, and metastasis [44]. The role of EZH2 in tumorigenesis was strengthened by its knockdown in both castrated xenograft mouse model and human PCa cells that led to androgen-independence growth arrest, significant reduction of tumour growth, and metastasis. The activity of EZH2 can also be regulated by other non-PRC2 independent pathways such as phosphatidylinositol-3-kinases (PI3K)/AKT signalling pathways. Dysregulation in PI3K/AKT signalling pathway is associated with perturbed cellular processes including apoptosis, cell growth, and survival, which in turn leads to tumour progression. PI3K/AKT signalling pathway switches the function of EZH2 from a PRC2 to a transcriptional coactivator of AR in PCa. Moreover, EZH2 may also methylate AR at lysines 630 and 632 and enhance its transcriptional activity without any synergistic relationship with polycomb repressors, suggesting that targeting more pathways including those that regulate the activity of EZH2 independent polycomb repressors would have therapeutic efficacy in suppressing PCa tumorigenesis and metastasis [44].

Other PRC2 binding partners that mediate aberrant epigenetic activities in PCa are bromodomain adjacent to zinc finger domain 2A (BAZ2A), JARID1A/B/D, and KDM4A. The BAZ2A, also known as TTF-1 interacting 5, represses numerous protein-coding genes that promote cell growth and proliferation. Overexpression of BAZ2A correlates with molecular subtype displaying a CpG island methylator phenotype that aberrantly alters gene expression, leading to PCa aggressiveness, metastasis, and recurrence [45]. BAZ2A harbours C-termini with tandem PHD finger/bromodomain that interacts with EZH2, which is a key mediator for heterochromatic histone signatures and* de novo* DNA methylation. Jumonji AT-rich interactive domain 1 protein (JARID1) is one of the JARID family proteins that partners with PRC2 to induce H3K4me2/3 through EZH2. JARID1 is also known to disrupt the activation of pathways such as Fe(II)/*α*-ketoglutarate-dependent oxygenase and regulates cell survival and metastasis. Overexpression of JARID1A/B/D has been observed in PCa and metastasis, and its deletion or knockdown is associated with poor prognosis [46]. The prognostic value of JARID1D in PCa was also observed when upregulated JARID1D repressed the transcription of* Snail family zinc finger 2* and* miR-21-targeted matrix metallopeptidase* (*MMP*) family genes, leading to reduced invasion and metastasis [47–49]. Regulation of AR-regulated and BTG2-targeted* miR-32* is associated with PCa chemoresistance [50]. Upregulation of JMJD2A, also known as lysine-specific demethylase 4A (KDM4A), has also been observed in both human and mice models with PCa. This significantly correlates with advanced PCa and metastasis. JMJD2A appears to interact with ETS transcription factor ETV1 as well as tumour suppressor genes phosphatase and tensin homolog (*Pten*) and yes associated protein 1 (YAP1), which promotes PCa initiation and aggressiveness [51]. Additionally, KDM4A was demonstrated to cooperate with miRNAs in regulating gene transcription and cellular processes in PCa, supporting an interaction between epigenetic alterations and miRNAs profiles [52].

## 6. MicroRNA and Epigenetics Alterations in Prostate Cancer

Noncoding miRNAs are evolutionary conserved short regulatory endogenous RNA molecules (~24 nucleotides in length) that are not translated into protein even though they are transcribed from DNA. They disrupt the function of messenger RNA, leading to dysregulation in RNA silencing and gene expression at posttranscriptional and translational levels. The miRNAs may function as tumour suppressors or oncogenes and can be either downregulated or upregulated in PCa. The miRNA profiling studies demonstrate that mRNAs may act independently or in partnership with other transcription factors to regulate gene transcription, which ultimately leads to perturbed cellular processes in PCa. Several* miRs* in addition to those described and reviewed by Lo et al. 2013 are dysregulated in PCa [53]. The* miR-101/31* was found to be downregulated in metastatic PCa, affecting the expression of EZH2 and regulation of PRC1 and B lymphoma Mo-MLV insertion region 1 homolog (BMI1) [54, 55]. This promotes tumour progression by targeting AR, Stathmin 1, and cyclooxygenase-2, which were shown to be an independent prognostic indicator for recurrent PCa [55, 56]. The* miR-24* was demonstrated to target proapoptotic gene Fas associated factor 1 by binding to its open reading frame, thereby enhancing apoptosis in DU-145 PCa cells [57]. In other studies, suppression of p63-mediated* miR-205*, Frizzled7-mediated* miR-613,* and* Myc*-mediated* miR-26a *expression levels was observed in PCa tissues as compared to their normal counterparts [24, 58]. Moreover, stable* miR-26a* induced G1 phase arrest and epithelial mesenchymal transition (EMT), leading to significantly reduced proliferation and metastasis via activation of Wnt5a pathway [58]. Similar effects in relation to EMT regulation were observed with downregulation of the* miR-182/203* via suppression of SNAI2 and with the* miR-144/145* associated with bone, skeletal, and seminal vesicle metastases in PCa patients [59, 60]. Genistein downregulates* miR-205/31*,* miR-221/222*,* miR-574-36b*, and* miR-574-36b* and confers resistance to chemotherapy-induced apoptosis in PCa cells [61–63]. Demethylation of* miR-146a* promoter by 5-Aza-2′-deoxycytidine significantly enhanced* miR-146a* expression, leading to delayed progression of castration-resistant PCa. [64].

## 7. Risk Factors for Prostate Cancer and Associated Epigenetics Alterations

**Figure 2:**
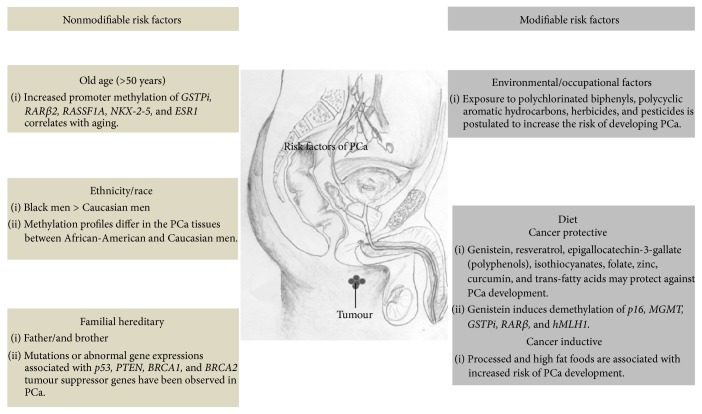
Risk factors associated with the development of prostate cancer. PCa risk factors can be categorised as nonmodifiable (e.g., old age, ethnicity/race, and familial hereditary) and modifiable (diet and environmental/occupational factors).

A significant amount of data has shown that certain risk factors including old age, familial hereditary, and ethnicity/race may induce PCa growth by influencing both genetic and epigenetic factors [65]. Advancing age and ethnicity are the greatest nonmodifiable risk factors and play a pivotal role in PCa development (Figure 2). The risk of developing PCa increases exponentially after the age of 50 years [66]. Seminal work published by Kwabi-Addu and his coauthors in the Clinical Cancer Research showed that methylation of* GSTPi*,* RARβ2*,* RASSF1A*,* NK2 homeobox 5 *(*NKX-2-5*), and* estrogen receptor 1 *(*ESR1*) tumours suppressor genes in prostate tissues is age-independent [67]. Methylation of these genes was shown to initiate in normal prostate tissues as old age strikes and markedly increases during the progression of PCa [67]. Age-related CpG island methylation is significantly enriched with DNA binding and transcriptional factors [68]. Jung et al. and his team in Van Andel Research Institute are currently investigating the epigenomic changes following environmental factors including aging. They developed the methylated CpG island recovery assay, which is a reliable method for comparing genome-wide DNA methylations in normal and cancer tissues [69]. More recently, the same group hypothesised that polycomb complex resulting from KDM2B interaction with PRC1/2 that recognize unmethylated CpG islands may degrade with age. This would, in turn, allow access of CpG islands to the DNMT3A and DNMT3B, leading to partial DNA methylation in aging men [70].

The risk of PCa is increased in black African and American men compared to Caucasian men, suggesting an important role of ethnicity/race in PCa development [71]. Several studies showed that black South African men present with higher PSA levels and more aggressive and metastatic PCa than black American men. The presence of these phenotypes correlates with socioeconomic status, poor PCa awareness, and screening facilities associated with developing countries [66, 71]. Racial/ethnic disparities in epigenetic alterations have been observed in PCa tissues and correlate with racial differences in cancer prognosis and survival [72]. Devaney et al. [73] revealed that genome-wide methylation profiles differ in the PCa tissues between African-American and Caucasian men. However, considerable research investigations are still required to elucidate the role of these methylation differences in PCa disparity.

Similar to most cancers, PCa is a genetic disease that can also be caused by both germline and somatic mutations. Family hereditary has been postulated to increase the risk of PCa development. Men older than 55 years who have a first-degree male relative (i.e., father, son, and brother) diagnosed with PCa are at higher risk of acquiring PCa [74]. Genetic susceptibility and aberrant epigenetics may predispose individuals to PCa [65]. For instance, mutations or abnormal gene expression associated with classic genes such as* p53*,* PTEN*,* BRCA1,* and* BRCA2* may contribute to PCa development and metastasis [75–77]. Epidemiological studies have also indicated that male carriers of* BRCA1 and BRCA2* mutations have the highest risk of developing PCa compared to noncarriers of these mutations. This coincides with more aggressive disease and low rates of survival [78, 79].

Diet and environmental and occupational factors are hypothesised to be modifiable PCa-related risk factors. However, the mechanisms underlying the link of these factors and PCa development remain unclear [80, 81]. The *α*-methylacyl-CoA racemase is a peroxisomal enzyme required for the oxidation of branched-chain trans-fatty acids from red meat and dairy products. This enzyme was found to be aberrantly upregulated in PCa cells, supporting the hypothesis that a diet high in saturated or trans fats is associated with increased risk for PCa development [82, 83]. Certain diets containing antioxidant or anticancer properties such as genistein, resveratrol, epigallocatechin-3-gallate (polyphenols), isothiocyanates, folate, zinc, curcumin, and mono- or polyunsaturated fatty acids may serve as protective factors for PCa (Figure 2). Some of these anticancer agents were found to inhibit tumour proliferation and promote apoptosis by suppressing trimethylation of H3K27 through PRC2 arrest [84, 85]. Genistein is an important nutraceutical compound derived from soy products and has been shown to confer protection from developing cancer. Genistein suppresses multiple cellular processes such as proliferation and angiogenesis by demethylating tumour suppressor genes* p16*,* MGMT*,* GSTPi*,* RARβ,* and* hMLH1*. Rajvir Dahiya and his colleagues from University of Southern California at San Francisco have recently demonstrated that genistein regulates various miRs including* miR-1260b*, leading to the upregulation of* sFRP1* and* Smad4* in PCa cell lines via DNA demethylation and histone modifications, suggesting that diet may also prompt epigenetic alterations and contribute to PCa development [86].

## 8. Epigenetic Therapy in Prostate Cancer

**Figure 3:**
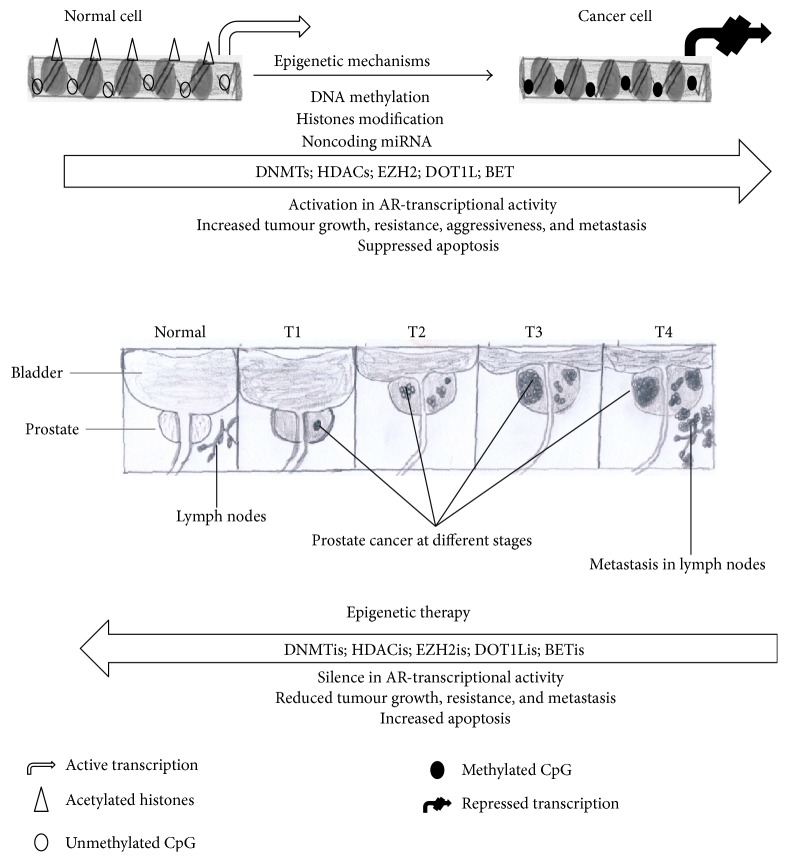
Epigenetic mechanisms and therapy in advanced prostate cancer. Prostate cancer follows aberrant epigenetic alterations that are associated with perturbed cellular processes that are critical in tumorigenesis. In normal cell, CpG islands are protected from DNA methylation and deacetylation whereas a prostate cancer cell is characterised by tumour growth, differentiation, resistance, and metastasis resulting from aberrant DNA methylation and deacetylation. AR: androgen receptor; BET: bromodomain and extraterminal; CpG: cytosine-phosphate-guanine island; DNMTs: DNA methyltransferases; DOT1L: DOT1-like histone H3K79 methyltransferase; EZH2: enhancer of zeste 1; HDACs: histone deacetylases; DNMTs: DNA methyltransferase inhibitors; HDACs: histone deacetylase inhibitors.

Substantial knowledge on abnormal epigenetic alterations and cancer development is now being translated into novel anticancer therapeutic approaches [87, 88]. Unlike genetic mutations, aberrant epigenetic alterations are potentially pharmaceutically reversible making them attractive targets for chemotherapeutic approaches. DNMTs, HDACs, EZH2, DOT1-like histone H3K79 methyltransferase (DOT1L), and bromodomain and extraterminal (BET) are the current targets of epigenetic therapies (Figure 3). Inhibitors against these epigenetic mediators emerged as promising chemotherapeutic agents in several cancer models [87, 89].

Available demethylating agents that inhibit the action of DNMT include nucleoside (5-azacytidine, 5-azacytidine-2′-deoxycytidine/decitabine, and zebularine) and nonnucleoside (procaine, procainamide, disulfiram, and RG108) inhibitors [88]. These inhibitors have exhibited efficient antitumorigenic activities by suppressing an altered expression of tumour suppressor genes in haematological malignancies, breast, gastric, lung, ovarian, and hepatocellular carcinomas. Convincing clinical evidence of most DNMT inhibitors in PCa is still lacking and remains elusive. However, the epigenetic therapeutic potential of decitabine, procainamide, disulfiram, and RG108 was recently observed in human PCa cell lines and xenograft models, correlating with significant reduction in tumour growth and increased apoptosis [87–91].

HDAC inhibitors (HDACis) are class of anticancer agents that reverse autonomous epigenetic alterations by disrupting the activity of HDACs, and their action coincides with sustained apoptosis resulting from impeded cell differentiation, angiogenesis, and metastasis. Vorinostat and romidepsin are the first FDA and EMEA approved HDACis and are currently used to treat uncontrolled cutaneous T cell lymphoma. Vorinostat has been investigated extensively in biologic, preclinical, and clinical studies involving several cancers and has emerged as a promising antitumor and antimetastatic agent with a favourable safety profile. Treatment with vorinostat represses PC-3 xenograft tumours and inhibits cell proliferation and metastasis in PC-3, LNCaP, and DU-145 human PCa cell lines [92]. Similar effects were observed in vorinostat-treated mice transplanted with CWR222 PCa tumours [93]. Romidepsin disrupts the interaction between Hsp90 and its binding partners, thereby inducing the* Hsp90* hyperacetylation and abrogation of AR signalling. In castration-resistant PCa clinical models, romidepsin correlates with minimal clinical activity. Other new HDACis that are currently undergoing investigation in PCa cell line models include panobinostat, MCL33(S)-2, and MHY219 [94, 95]. Combination therapy of vorinostat and romidepsin with other chemotherapeutic drugs including demethylating agents was shown to have more maximal activity than monotherapy and synergistically enhanced apoptosis in PCa cell lines ALVA-31, LNCaP, and DU-154 [96, 97]. Recently, a new hybrid of vorinostat and a topoisomerase inhibitor WJ3543 exhibited potential antitumor activities, which overlapped with repressed cell growth, proliferation, and increased apoptosis in metastatic PCa [98].

While the efficacy of most lysine methyltransferases inhibitors in PCa remains obscure, there has been some advancement in preclinical development and phase 1 clinical studies with inhibitors targeting EZH2, DOT1L, and BET. BET inhibitor I-BET762 potentially suppressed cell growth and tumour aggressiveness* in vivo* by reducing* MYC* expression in PCa cancer cell lines, xenograft mouse model, and human tissues [99, 100]. EZH2 inhibitors GlaxoSmithKline-126 (GSK-126) and 3-deazaneplanocin-A reduced AR-transcriptional activity, leading to the inhibition of cancer cells self-renewal mechanisms, tumorigenesis, and metastasis mediated by aberrant EZH2 upregulation in PCa cells [101, 102]. Furthermore, combinations of nontoxic topoisomerase poisons VP-16 and GSK-126 significantly enhanced apoptosis in* in vitro* viability assays [102]. Other EZH2 inhibitors such as EPZ005667, GSK343, EP2-6436, Novartis, and EI1 also demonstrated their chemotherapeutic potential in treating several malignancies. Anti-DOT1L inhibitors such as EPZ004777, EPZ003696, EPZ5676, Yao CMP4, BrSAH, and SGC946 have been identified and tested in leukemic cells, where they were associated with suppression of H3K27 and tumour growth [103–107]. These inhibitors and other EZH2 inhibitors including EPZ005667, GSK343, EP2-6436, Novartis, and EI1 warrant clinical investigations in PCa.

## 9. Conclusion

Current evidence supports previous studies and demonstrates that epigenetic mechanisms play a critical role in PCa development. PCa-related epigenetic alterations may be triggered by induced genomic instabilities as well as various modifiable and nonmodifiable risk factors. Aberrant DNA methylation, histone modification, and noncoding RNA are tightly linked and synergistically alter gene transcription and normal gene function. These mechanisms lead to perturbed cellular pathways that are associated with abnormal or pathological phenotypes in prostate gland. Although several transcriptional factors have been demonstrated to interact with epigenetic regulators and modifiers, the underlying mechanisms are clearly complex and remain elusive. In terms of clinical application, epigenetic alterations in PCA are also potentially pharmacologically reversible. However, more preclinical and clinical studies are still needed to explore the chemotherapeutic potential of demethylation agents including EZH2, DOT1L, and BET. This may enable the development of more novel chemotherapeutic drugs that will be used in conjunction with current conventional treatments such as ^77^Lu-PSMA-RLT to improve the management of refractory and metastasised PCa.
